# Oral health-related quality of life, impaired physical health and orofacial pain in children and adolescents with juvenile idiopathic arthritis – a prospective multicenter cohort study

**DOI:** 10.1186/s12903-023-03510-0

**Published:** 2023-11-20

**Authors:** Josefine M. Halbig, Birgitta Jönsson, Elisabeth G. Gil, Anne N. Åstrøm, Veronika Rypdal, Paula Frid, Thomas A. Augdal, Johannes Fischer, Lena Cetrelli, Marite Rygg, Anette Lundestad, Karin Tylleskär, Ellen Nordal, For the Norwegian JIA Study – Temporo-mandibular Involvement, Oral Health, Uveitis, Bone Health, Quality of Life in Children with Juvenile Idiopathic Arthritis, Karen Rosendahl, Marit Slåttelid Skeie, Ellen Nordal, Annika Rosén, Elisabeth G. Gil, Johannes Fischer, Xieqi Shi, Oskar Angenete, Gunnar Lyngstad, Marie Sager, Astrid J. Feuerheim, Thomas A. Augdal, Josefine M. Halbig, Athanasia Bletsa, Marit Midtbø, Larissa von Wangenheim Marti, Mats Säll, Keijo Luukko, Marianne Lothe Vollan, Erik Haro, Tone Kvinnsland Amdal, Susanne Irene Tobiesen Eidset, Line Rapp Simonsen, Marte Grimsmo Teige, Brita Lena Hansen, Lisbeth Aune

**Affiliations:** 1 Public Dental Health Competence Centre of Northern Norway (TkNN), Tromsø, Norway; 2 Research Group Child and Adolescent Health, Department of Clinical Medicine, Faculty of Health Sciences, UiT - The Arctic University of Norway, Tromsø, Norway; 3 Department of Periodontology, Institute of Odontology, University of Gothenburg, Gothenburg, Sweden; 4 Department of Clinical Dentistry, University of Bergen, Bergen, Norway; 5 Department of Pediatrics, University Hospital of North Norway, Tromsø, Norway; 6 Department of Otorhinolaryngology, Division of Oral and Maxillofacial Surgery, University Hospital of North Norway, Tromsø, Norway; 7 Department of Clinical Dentistry, UiT - The Arctic University of Norway, Tromsø, Norway; 8 Section of Pediatric Radiology, University Hospital of North Norway, Tromsø, Norway; 9 Center of Oral Health Services and Research (TkMidt), Trondheim, Norway; 10 Department of Clinical and Molecular Medicine, Norwegian University of Science and Technology (NTNU), Trondheim, Norway; 11 Department of Pediatrics, St. Olavs Hospital, Trondheim, Norway; 12 Department of Pediatrics, Haukeland University Hospital, Bergen, Norway

**Keywords:** Juvenile idiopathic arthritis, Oral health, Quality of life, Orofacial pain, Adolescent, Child

## Abstract

**Background:**

Knowledge on oral health-related quality of life (OHRQoL) in children and adolescents with juvenile idiopathic arthritis (JIA) is limited, and longitudinal studies are lacking. We aimed to describe OHRQoL in children and adolescents with JIA compared to controls, and to explore the validity and internal consistency of the Early Childhood Oral Health Impact Scale (ECOHIS) and the Child Oral Impact on Daily Performance (Child-OIDP). Furthermore, we wanted to investigate associations between OHRQoL and orofacial pain, physical health, disease activity, and temporomandibular joint (TMJ) involvement in JIA.

**Methods:**

The Norwegian prospective, multicenter cohort study recruited participants with JIA between 4 and 16 years of age and corresponding controls from three pediatric university hospital departments and public dental health services. In the present study, we analyzed OHRQoL in all children < 12 years with the ECOHIS and adolescents ≥ 12 years with the Child-OIDP at the first visit and the two-year follow-up. Associations between OHRQoL and JIA characteristics, collected in clinical exam and questionnaires, were analyzed in logistic regressions.

**Results:**

The same OHRQoL questionnaire was completed both at first visit and two-year follow-up in 101 children < 12 years (47 JIA, 54 controls) and 213 adolescents ≥ 12 years (111 JIA, 102 controls). The frequency of OHRQoL impacts in children was similar at the first visit and the two-year follow-up (ECOHIS > 0: JIA group 81% and 85%, *p* = *0.791*; control group 65% and 69%, *p* = *0.815*), while adolescents with JIA reported fewer impacts at the two-year follow-up (Child OIDP > 0: JIA group 27% and 15%, *p* = *0.004;* control group 21% and 14%, *p* = *0.230*). The internal consistency of the OHRQoL instruments was overall acceptable and the criterion validity indicated that the instruments were valid at both visits. Orofacial pain was more frequent in children and adolescents with JIA than in controls. We found associations between OHRQoL impacts and orofacial pain, impaired physical health, disease activity, and TMJ involvement.

**Conclusions:**

Children and adolescents with orofacial pain or impaired physical health were more likely to report impacts on daily life activities than those without. Pediatric rheumatologists and dentists should be aware of impaired OHRQoL in individuals with JIA with active disease or temporomandibular joint involvement.

**Trial registration:**

Registered on clinicaltrials.gov (NCT03904459, 05/04/2019).

**Supplementary Information:**

The online version contains supplementary material available at 10.1186/s12903-023-03510-0.

## Background

Children and adolescents with juvenile idiopathic arthritis (JIA) have a chronic rheumatic disease with onset before the age of 16 years and several different characteristics and symptoms due to joint inflammation [1]. In Norway the annual incidence rate is between 14 and 23 per 100,000 under 16 years of age, which is somewhat higher than corresponding rates from other geographical regions, but comparable to other Scandinavian countries [2–5]. Despite the increasing availability of biologic disease-modifying antirheumatic drugs in recent decades, there is still a substantial proportion of patients with active disease in long-term follow-up of JIA cohorts [6, 7]. Children and adolescents with JIA report a significant impairment of health-related quality of life (HRQoL) with impacts on daily functioning both at home and in school settings [8, 9].

Oral health is not only the absence of pathological findings in the oral cavity but should incorporate the patient’s perceptions, such as “the ability to speak, smile, smell, taste, touch, chew, swallow, and convey a range of emotions through facial expressions with confidence and without pain, discomfort, and disease of the craniofacial complex” [10], in other words, the patient’s oral health-related quality of life (OHRQoL). Research regarding OHRQoL in children and adolescents with JIA is limited and needs further exploration [11]. A small study on oral health and OHRQoL reported no differences in OHRQoL in children and adolescents with JIA, compared to controls [12], while three recent studies on orofacial symptoms [13, 14] and TMJ arthritis [15] found that self-reported orofacial symptoms and TMJ arthritis have a negative impact on OHRQoL [13, 15]. One study found that 22% of the children and adolescents were severely impacted by orofacial symptoms and dysfunction [14]. To our knowledge there is no longitudinal study on OHRQoL in JIA.

Temporomandibular joint (TMJ) arthritis is a common finding in children and adolescents with JIA that can occur throughout the disease course [9, 16, 17]. Arthritis in the TMJ may lead to severe craniofacial growth disturbances, functional problems, and pain in the orofacial region, which may impact overall oral health [18, 19]. There is limited research on oral health in children and adolescents with JIA, and the findings are divergent [11]. Dental plaque and gingival bleeding, as an indicator of gingival inflammation, are more commonly reported clinical findings in children and adolescents with JIA than in controls [11, 20, 21]. Caries prevalence does not significantly differ in children with and without JIA, according to recent reports [11, 22].

In order to improve the OHRQoL in children and adolescents with JIA, it is important that both dental and medical practitioners are aware of the implications of JIA on both OHRQoL and the family dynamics [23, 24]. Thus, the overall aim of this study was to gain further knowledge on such implications.

In detail, the aims of this study were first to describe oral health-related conditions and OHRQoL in a cohort of children and adolescents with JIA compared to controls without JIA at the time of inclusion (the first visit) and after two years (the two-year follow-up). Secondly, to explore the internal consistency and validity of the Early Childhood Oral Health Impact Scale (ECOHIS) and the Child Oral Impact on Daily Performance (Child-OIDP) in our JIA cohort. Thirdly, to investigate associations between OHRQoL and orofacial pain and impaired physical health among children and adolescents with and without JIA; and fourthly, to investigate associations between OHRQoL and disease activity and TMJ involvement in the JIA cohort.

## Methods

### Study design and population

This study is part of the Norwegian JIA study (NorJIA, www.norjia.com). NorJIA is a prospective, longitudinal multicenter cohort study with a comparative design. We collected data at the first visit and after two years in 224 children and adolescents with JIA and a corresponding control group without JIA between April 2015 and October 2020. At the university hospitals in Bergen, Trondheim, and Tromsø, pediatric rheumatologists invited participants diagnosed with JIA according to the International League of Associations for Rheumatology (ILAR) criteria [25]. The control group, children and adolescents without JIA listed for routine examination in public dental health service care, were recruited from both rural and urban areas; the University Dental Clinic in Bergen, Public Dental Health Clinics in and around Bergen (Western Norway), the Public Dental Health Clinic in Stjørdal (Central Norway) and the University Dental Clinic in Tromsø (Northern Norway). They were matched 1:1 at each center to the participants with JIA, according to age and gender. Both at the time of inclusion (the first visit) and after two years (the two-year follow-up) the participants underwent the same examinations and answered the age-appropriate questionnaires. All questionnaires were completed either before the oral examination or closely after the examination with a maximum of two reminders. This report includes the children and adolescents who answered the same OHRQoL instrument at both visits. The sample size calculation for our cohort was based on caries estimates described by Gil et al. [22].

### Oral health questionnaire

Adolescents ≥ 12 years of age and parental proxies of children younger than 12 years completed a questionnaire including sociodemographic data and oral health information. For this report, we dichotomized the educational level of caregivers (university/college = 0, primary school/high school = 1), the number of caregivers (two caregivers = 0, one caregiver = 1), the frequency of toothbrushing (twice a day or more = 0, less than twice a day = 1) and use of dental floss (several times weekly or more = 0, less than several times weekly = 1), self-reported clinical signs such as gingival bleeding during toothbrushing (never = 0, sometimes or more = 1), pain or discomfort during tooth brushing (no = 0, yes = 1), and the frequency of intraoral ulcerations (less than once a year = 0, several times yearly = 1). The original and new codes are presented in the supplemental material (Supplemental, Additional file S1, Table 1).

### Oral examinations

The oral examinations were carried out by six experienced dentists, who underwent training and calibration exercises both before and during the study period, as described by Gil et al. [20, 22]. Caries, at both the enamel and dentin levels (d_1-5_f /D_1-5_F), was diagnosed on all five surfaces of the second primary or the first permanent molars by visual inspection and on bitewing radiographs as described elsewhere [22] and were dichotomized as 0 = absent (d_1-5_f/D_1-5_F = 0) or 1 = present (d_1-5_f /D_1-5_F > 0). The missing component was not included in the d_1-5_f /D_1-5_F- index due to only a few missing teeth. For adolescents, supragingival debris and calculus were measured according to the Simplified Oral Hygiene Index (OHI-S) by Greene and Vermillion [26] and gingival bleeding was assessed according to the Gingival Bleeding Index (GBI) by Ainamo and Bay [27] modified as described elsewhere [20]. The OHI-S was dichotomized to 0 = good oral hygiene, representing approximately 33% of the participants with the lowest plaque and calculus scores (OHI-S cut off 0.5) and 1 = moderate to poor oral hygiene with OHI-S ≥ 0.5. The GBI was dichotomized with a cutoff of 10% bleeding: 0 = no or less than 10% bleeding points and 1 = 10% or more bleeding points.

### Early childhood oral health impact scale (ECOHIS)

To assess the impacts of oral health problems on quality of life in children (defined as participants < 12 years of age in this report), we used the ECOHIS, a questionnaire with 13 items answered by a parental proxy [28], previously validated in a general local population of Norwegian children [29]. The response options were recoded on a Likert scale with scores from 0 = *never* to 4 = *very often* (Supplemental, Additional file S1, Table 2), where missing single items were replaced by the mean score of the section. Questionnaires with more than two missing items in the child section or more than one missing item in the family section were excluded as described elsewhere [28, 30, 31]. The scores on the 13 items were added to the total ECOHIS additive (ADD) score (range 0—52). To calculate changes in OHRQoL over time, the two-year follow-up score was subtracted from the first visit score, forming the ECOHIS change ADD score, where negative values indicated worsened and positive values improved precepted oral health at the two-year follow-up [32]. Additionally, the score from the first visit and the two-year follow-up was dichotomized to 0 = no impacts and 1 = ADD score > 0 when at least one ECOHIS item was affected.

### Child oral impact on daily performance index (Child OIDP)

For adolescents (defined as participants ≥ 12 years of age in this report), OHRQoL was assessed using the Child-OIDP [33], an eight-item instrument adapted from the adult OIDP inventory [34] and validated in a general local population of Norwegian adolescents [29]. The adolescent was asked by the dentist, prior to the oral examination, whether each of the eight daily activities was affected by problems with their teeth or mouth during the last three months. The response options were coded on a Likert scale with the original scores *never* = 0, *once or twice a month* = 1, *once or twice a week* = 2, and *every day/almost every day* = 3. The eight scores were added to the Child-OIDP ADD score (range 0—24). The ADD score was dichotomized to 0 = no impacts and 1 = at least one impact in the last three months. A Child-OIDP change ADD score was calculated as described for the ECOHIS.

### Global oral health outcomes as reported by adolescents and parental proxies

Adolescents and parental proxies of children were asked to rate their/their child’s dental health and tooth appearance. They were asked, *“How would you rate your dental health?”*. The response options were coded on a five-point Likert scale and dichotomized into good = 0 including the original responses *very good* and *good* (scores 1 and 2) and poor = 1 including *neither nor*, *poor,* and *very poor* (scores 3–5). Second, they were asked, *“How satisfied are you with the appearance of your teeth?”*. The five response options were dichotomized into satisfied = 0 including *very satisfied* and *satisfied* (scores 1 and 2) and unsatisfied = 1 including *neither nor*, *unsatisfied*, *and very unsatisfied* (scores 3–5).

### Orofacial pain

In accordance with the international consensus-based recommendations for orofacial assessment in patients with JIA [35], all participants were asked during the orofacial examination visits, if they ever had pain in their jaw, temple, ear, or in front of the ear on at least one side. Those answering “yes” were also asked, which of the three alternatives best described a possible pain in one of the regions during the last 30 days. The three answer options were dichotomized to no pain = 0 (the original answer *no pain*) and pain = 1 including *pain comes and goes* and *pain all the time*.

### Child health questionnaire

The parent-administered Norwegian version of the child health questionnaire (CHQ-PF50) [36] is a generic measure of the physical and psychosocial health of children and adolescents [37]. The CHQ physical summary score (PhS) ranges from 0–100 (mean 50, SD ± 10), with higher scores indicating a better status and health [37, 38]. The PhS was dichotomized as 0 = normal with scores ≥ 40 and 1 = impaired with scores < 40, accordingly more than one SD lower than the mean of the US norm population [37].

### JIA characteristics

The pediatric rheumatologists collected clinical characteristics, including age at disease onset, disease duration, JIA category according to the ILAR criteria, the number of active joints, the cumulative number of affected joints, and previous and current disease-modifying antirheumatic drugs (DMARDs). Additionally, the pediatric rheumatologists rated the JIA disease activity on a visual analog scale (VAS) from 0–10 (MDgloVAS: 0 = “no activity”, 10 = “high activity”). The patients (children ≥ 9 years) or proxies (children < 9 years) reported functional ability in the Childhood Health Assessment Questionnaire (CHAQ), as well as the disease impact on overall wellbeing (PRgloVAS) and pain during the last week on a VAS (range 0–10, 0 = “no influence”/”no pain” and 10 = “severely influenced”/ “severe pain”). MDgloVAS, PRgloVAS, and global pain reports were dichotomized to “0″ for those with a rating of 0 and “1″ for ratings > 0 on the VAS scale. We analyzed C-reactive protein (CRP) and erythrocyte sedimentation rate (ESR). The juvenile arthritis disease activity score (JADAS71) was calculated based on the MDgloVAS, the PRgloVAS, the active joint count, and the ESR (CRP with missing ESR), as appropriate [39]. For analysis, we dichotomized to 0 = JADAS < 1 and 1 = JADAS ≥ 1 as an indicator for active disease for all JIA categories. Former clinical TMJ involvement and clinical TMJ involvement at the study visits were registered by the pediatric rheumatologists based on the best judgment of all available information, including symptoms, clinical examination, and radiologic evaluation up to the study visit [40].

### Statistical methods

All statistical analyses were conducted using STATA version 17 software (STATA Corp., College Station, Texas, USA). Means, 95% confidence intervals, medians, interquartile ranges, and frequencies were reported for descriptive data. Chi-squared- and Fisher’s exact-test were applied, to evaluate differences in frequencies of OHRQoL impacts between groups at one timepoint. Mann–Whitney U-tests were applied, to evaluate differences of OHRQoL mean ADD scores between groups. McNemar’s test and Wilcoxon signed-rank test were used, to assess change within the frequencies of OHRQoL impacts and OHRQoL mean ADD scores in the respective group over time. Effect sizes for the OHRQoL mean ADD scorers were calculated for differences between JIA and control group at one timepoint by Cohen’s d for two independent samples using groups (JIA/Controls):

$$\frac{mean\;JIA\;group-mean\;Controls}{SD\;(mean)\;pooled}$$

For differences between first visit and two-year follow-up within one group Cohen’s d was calculated according to the following formula:

$$\frac{first\;visit\;mean-follow-up\;mean}{SD(first\;visit)}$$

Effect sizes of ≤ 0.2 were considered small, > 0,2 and ≤ 0.5 medium and ≥ 0.8 large [41].To assess the internal consistency reliability of the two OHRQoL instruments, we used Cronbach’s alpha, and to assess the validity, we compared the mean OHRQoL ADD scores according to the two categories of global oral health outcomes (“good” *vs.* “poor oral health” and “satisfied” *vs.* “unsatisfied with tooth appearance”, Mann–Whitney U-test). To assess the longitudinal validity of the OHRQoL instruments, we evaluated the association between OHRQoL change ADD scores and change in the category of the reference variables, in terms of the global oral health outcomes and self-reported clinical signs, using one-way ANOVA and Bonferroni post hoc test. To analyze associations between OHRQoL and orofacial pain, impaired overall physical health, JIA disease activity, and TMJ involvement, we applied logistic regression both unadjusted and adjusted for age, gender, parental educational level, and group affiliation (JIA/Controls).

## Results

### Study population

**Fig. 1:**
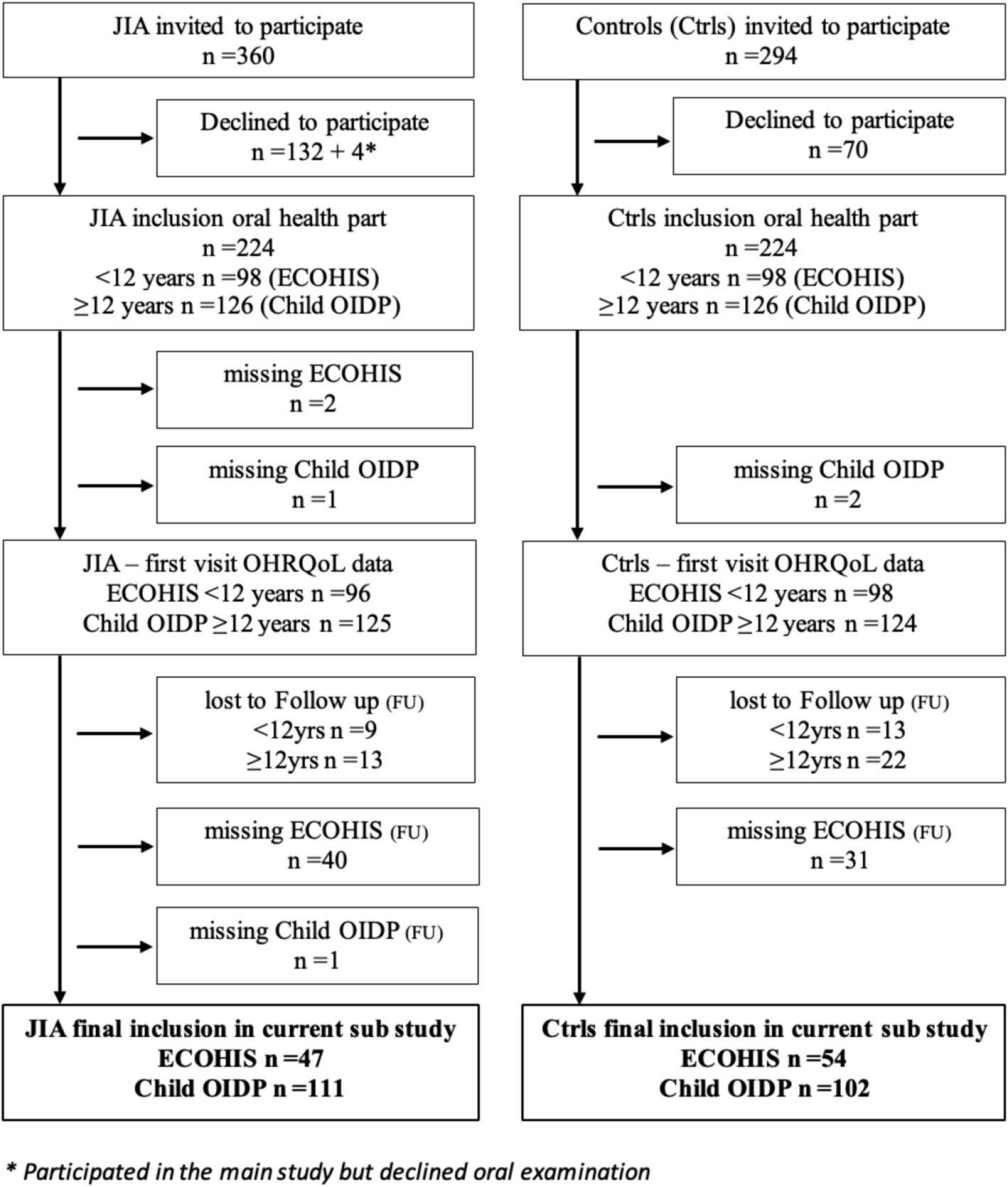
Flow diagram of participating children and adolescents with juvenile idiopathic arthritis (JIA) and controls

Of the 360 invited patients with JIA and 294 controls, the 47 children and 111 adolescents with JIA and 54 children and 102 adolescents without JIA, who completed the same OHRQoL instrument at both visits, were included in this study (Fig. 1). The distribution of sociobehavioral characteristics, parent/patient-reported oral symptoms, and clinical oral findings among children (< 12 years of age) and adolescents (≥ 12 years of age) are presented in Tables 1 and 2, respectively, together with JIA disease characteristics for the JIA group only.

**Table 1:** Distribution of characteristics among children with JIA and controls at both visits

Variable	Children—First Visit					Children—Two-Year Follow-up				
	**JIA**		**Controls**			**JIA**		**Controls**		
	%	(n/N)	%	(n/N)	*p-value*^a^	%	(n/N)	%	(n/N)	*p-value*^a^
Median age at visit, years (IQR)	7.6	(6.7–8.9)	7.8	(6.8–9.1)		9.7	(8.6–10.9)	9.8	(8.9–11.0)	
Female gender	70.2	(33/47)	75.9	(41/54)						
Toothbrushing, twice a day or more	68.1	(32/47)	75.9	(41/54)	*0.380*	71.7	(33/46)	82.7	(43/52)	*0.195*
Tooth flossing, several times a week or more	10.6	(5/47)	11.3	(6/53)	*1.000*	17.8	(8/46)	11.8	(6/51)	*0.431*
Gingival bleeding during toothbrushing, sometimes or more	43.5	(20/46)	33.3	(18/54)	*0.298*	50.0	(23/46)	33.3	(17/51)	*0.096*
Pain or discomfort during toothbrushing	6.4	(3/47)	1.9	(1/54)	*0.336*	8.7	(4/46)	0	(0/51)	***0.047***
Ulcerations, several times yearly or more	31.1	(14/45)	13.0	(7/54)	***0.028***	32.6	(15/46)	16.0	(8/50)	*0.057*
Orofacial pain ever	27.7	(13/47)	11.1	(6/54)	***0.034***	31.9	(15/47)	5.6	(3/54)	***0.001***
Orofacial pain last 30 days	14.9	(7/47)	1.9	(1/54)	***0.024***	21.3	(10/47)	3.7	(2/54)	***0.011***
Caries, d_1-5_f/D_1-5_F-level > 0	26.1	(12/46)	27.8	(15/54)	*0.849*	37.0	(17/46)	33.3	(18/54)	*0.243*
JIA disease characteristics										
Median age at disease onset, years (IQR)	2.4	(1.8–4.8)								
Disease duration, median years (IQR)	4.5	(3.1–5.9)				6.5	(5.1–7.8)			
Polyarticular disease course (> 4 affected joints)	48.9	(23/47)				48.9	(23/47)			
Oligoarticular JIA category^1^	51.1	(24/47)				51.1	(24/47)			
DMARDs ongoing	72.3	(34/47)				66.0	(31/47)			
Active joints > 0	17.0	(8/47)				10.6	(5/47)			
MDglobalVAS > 0	34.0	(16/47)				21.7	(10/46)			
ESR ≥ 20 mm/h	2.2	(1/46)				2.1	(1/47)			
PRglobalVAS > 0	72.3	(34/47)				61.7	(29/47)			
VAS global pain > 0	51.1	(24/47)				74.5	(35/47)			
CHAQ > 0	66.0	(31/47)				59.6	(28/47)			
JADAS ≥ 1	70.2	(33/47)				63.0	(29/46)			
TMJ involvement ever^2^	23.4	(11/47)				27.7	(13/47)			
TMJ involvement at study visit^2^	4.3	(2/47)				2.1	(1/47)			

Differences between JIA and Control group: ^a^chi-square test/Fisher’s exact

^1^JIA category – *oligo arthritis* (including persistent *n* = 19 and extended *n* = 5) vs non oligo arthritis (including systemic onset *n* = 2, polyarthritis RF^−^*n* = 12, polyarthritis RF^+^*n* = 0, psoriatic JIA *n* = 2, enthesitis related arthritis *n* = 2 and undifferentiated *n* = 5.

^2^TMJ involvement as recorded clinically by the pediatric rheumatologist

*JIA* Juvenile idiopathic arthritis, *IQR* interquartile range, *n/N* number observed/total number assessed for each variable, excluding missing values (total participants *N* = 47 JIA and *N* = 54 Controls), *d*_*1-5*_*f/D*_*1-5*_*F*  decayed and filled deciduous/ permanent teeth, enamel caries included, *DMARDs* disease-modifying antirheumatic drugs (synthetic and/or biologic agents), *MDglobalVAS* Physician's global assessment of disease activity, *ESR* Erythrocyte sedimentation rate, *VAS* Visual analog scale, *PRglobalVAS* Parent's global assessment of disease activity, *CHAQ* Childhood Health Assessment Questionnaire, *JADAS* juvenile arthritis disease activity score, *TMJ* temporomandibular joint. *P-*values < 0.05 are marked in bold

**Table 2:** Distribution of characteristics among adolescents with JIA and controls at both visits

Variable	Adolescents—First Visit					Adolescents—Two-Year Follow-up				
	**JIA**		**Controls**			**JIA**		**Controls**		
	%	(n/N)	%	(n/N)	*p-value*^a^	%	(n/N)	%	(n/N)	*p-value*^a^
Median age at visit, years (IQR)	14.4	(13.3–15.3)	14.7	(13.3–15.5)		16.4	(15.3–17.4)	16.6	(15.2–17.5)	
Female gender	53.2	(59/111)	54.9	(56/102)						
Toothbrushing, twice a day or more	79.1	(83/105)	75.5	(77/102)	*0.541*	82.5	(85/103)	71.3	(72/101)	*0.057*
Tooth flossing, several times a week or more	22.1	(23/104)	22.6	(23/102)	*0.940*	25.5	(26/102)	22.8	(23/101)	*0.651*
Gingival bleeding during toothbrushing, sometimes or more	61.5	(64/104)	58.8	(60/102)	*0.691*	59.8	(61/102)	62.4	(63/101)	*0.707*
Pain or discomfort during toothbrushing	10.7	(11/103)	13.7	(14/102)	*0.505*	11.8	(12/102)	10.9	(11/101)	*0.844*
Ulcerations, several times yearly or more	25.0	(25/100)	36.3	(37/102)	*0.082*	34.7	(35/101)	40.6	(41/101)	*0.384*
Orofacial pain ever	42.7	(47/110)	14.7	(15/102)	** < *0.001***	38.7	(43/111)	16.7	(17/102)	** < *0.001***
Orofacial pain last 30 days	28.2	(31/110)	2.9	(3/102)	** < *0.001***	24.3	(27/111)	5.9	(6/102)	** < *0.001***
Caries—D_1-5_F-level > 0	52.3	(56/107)	57.8	(59/102)	*0.424*	57.3	(63/110)	59.8	(61/102)	*0.709*
Dental plaque and Calculus – OHI-S ≥ 0.5	76.3	(71/93)	54.1	(53/98)	***0.001***	75.5	(80/106)	63.5	(61/96)	*0.065*
Gingival bleeding – GBI ≥ 10%	63.6	(70/110)	51.0	(52/102)	*0.062*	59.3	(64/108)	37.8	(37/98)	***0.002***
JIA disease characteristics										
Median age at disease onset, years (IQR)	9.6	(3.6–11.7)								
Disease duration, median years (IQR)	4.9	(2.7–10.5)				6.9	(4.7–12.5)			
Polyarticular disease course (> 4 affected joints)	51.4	(57/111)				53.2	(59/111)			
Oligoarticular JIA category^1^	41.4	(46/111)				40.5	(45/111)			
DMARDs ongoing	64.0	(71/111)				62.2	(69/111)			
Active joints > 0	28.8	(32/111)				14.4	(16/111)			
MDglobalVAS > 0	37.8	(42/111)				29.4	(32/109)			
ESR ≥ 20 mm/h	4.5	(5/111)				2.7	(3/111)			
PRglobalVAS > 0	72.9	(78/107)				63.8	(67/105)			
VAS global pain > 0	62.6	(67/107)				59.1	(62/105)			
CHAQ > 0	53.3	(57/107)				47.6	(50/105)			
JADAS ≥ 1	62.6	(67/107)				61.2	(63/103)			
TMJ involvement ever^2^	35.1	(39/111)				36.9	(41/111)			
TMJ involvement at study visit^2^	12.6	(14/111)				7.2	(8/111)			

Differences between JIA and Control group: ^a^chi-square test/Fisher’s exact

^1^JIA category – *oligo arthritis* (including persistent *n* = 36 and extended *n* = 10) vs non oligo arthritis (including systemic onset *n* = 1, polyarthritis RF^−^*n* = 25, polyarthritis RF^+^*n* = 2, psoriatic JIA *n* = 3, enthesitis related arthritis *n* = 17 and undifferentiated *n* = 17.

^2^TMJ involvement as recorded clinically by the pediatric rheumatologist

*JIA* juvenile idiopathic arthritis, *IQR* interquartile range, *n/N* number observed/total number assessed for each variable, excluding missing values (total participants *N* = 111 JIA and *N* = 102 Controls). D_1-5_F = decayed and filled permanent teeth, enamel caries included. DMARDs = disease-modifying antirheumatic drugs (synthetic and/or biologic agents). MDglobalVAS = Physician's global assessment of disease activity. ESR = Erythrocyte sedimentation rate. VAS = Visual analog scale. PRglobalVAS = Parent's global assessment of disease activity. CHAQ = Childhood Health Assessment Questionnaire. JADAS = juvenile arthritis disease activity score. TMJ = temporomandibular joint. *P*-values < 0.05 are marked in bold

Dropout analyses, performed separately for children and adolescents with and without JIA, are presented in the supplementary files (Supplemental, Additional file S2, Tables 1 and 2). Children, included in this study, were significantly younger than those, who were lost to follow-up or excluded. For adolescents, this age difference was only significant in the JIA group. Additionally, adolescents with JIA, who were lost to follow-up, had significantly more caries at the D_1-5_F-level than those who participated in the two-year follow-up.

We found similar sociodemographic and behavioral characteristics in both the JIA and control group, except for lower paternal educational levels in adolescents with JIA than in controls (university/college education in 39.6% *vs.* 63.2%, p < 0.001). When controlling the association between OHRQoL impacts > 0 and JIA group affiliation (JIA/no JIA) for the parental educational level, we did not find significant changes in the results (ordinal logistic regression, results not shown).

### Oral health related conditions and OHRQoL

Compared to controls, more children with JIA reported oral ulcerations at least several times a year at the first visit, and pain or discomfort during toothbrushing at the two-year follow-up (Table 1). In the clinical examination, we found significantly more adolescents with JIA who had moderate to poor oral hygiene than controls at the first visit, and significantly more adolescents with JIA than controls who presented with gingival bleeding at the two-year follow-up (Table 2).

More than 80% of the children with JIA and more than 60% of the controls reported negative OHRQoL impacts (ECOHIS > 0), as shown together with the mean ECOHIS ADD scores for the JIA and control group in Table 3. We found higher mean ECOHIS ADD scores for children with JIA both at the first visit and the two-year follow-up with moderate effect sizes (ES 0.48 and 0.53, Table 3). The ECOHIS ADD scores for the first visit and the two-year follow-up did not differ significantly either in the JIA group or in the control group (Table 3). We did not find gender differences in frequencies or mean ADD scores for children with or without JIA (results not shown). When analyzing and comparing each separate item, more children with JIA reported difficulty eating some foods compared to the children in the control group at the first visit (31.9% *vs.* 13.0%, *p* = *0.021,* results not shown). At the two-year follow-up, more children with JIA reported having *pain in their teeth, mouth, or jaw* compared to children in the control group (74.5% *vs.* 51.9%, *p* = *0.019*). Additionally, more children with JIA reported having *missed preschool or school* because of dental problems or treatments (38.3% *vs.* 11.1%, *p* = *0.001*).

**Table 3:** OHRQoL impacts and mean OHRQoL ADD scores for both visits

**Variable**	**First Visit**	**Two-Year Follow-up**	*p-value*	*Effect size*^*e*^
Children < 12 years				
ECOHIS > 0, % (n/N)				
JIA	80.9 (38/47)	85.1 (40/47)	*0.791*^*b*^	
Controls	64.8 (35/54)	68.5 (37/54)	*0.815*^*b*^	
*p-value*	*0.073*^*a*^	*0.051*^*a*^		
Mean ECOHIS ADD score (SD)[95%CI]				
JIA	3.7 (4.2) [2.5–4.9]	4.7 (4.6) [3.3–6.0]	*0.134*^*d*^	*-0.23*
Controls	2.1 (2.7) [1.3–2.8]	2.6 (3.1) [1.7–3.4]	*0.304*^*d*^	*-0.20*
*p-value*	***0.027***^***c***^	***0.010***^***c***^		
*Effect size*^*f*^	*0.48*	*0.53*		
Adolescents ≥ 12 years				
Child OIDP > 0, % (n/N)				
JIA	27.0 (30/111)	15.3 (17/111)	***0.004***^***b***^	
Controls	20.6 (21/102)	13.7 (14/102)	*0.230*^*b*^	
*p-value*	*0.271*^*a*^	*0.742*^*a*^		
Mean child OIDP ADD score (SD)[95%CI]				
JIA	1.2 (2.5) [0.7–1.6]	0.5 (1.6) [0.2–0.8]	***0.033***^***d***^	*0.24*
Controls	0.5 (1.4) [0.2–0.8]	0.3 (0.8) [0.1–0.4]	***0.040***^***d***^	*0.19*
*p-value*	*0.160*^*c*^	*0.608*^*c*^		
*Effect size*^*f*^	*0.31*	*0.22*		

^a^Chi2/Fisher’s exact – test (differences in frequencies between groups at one timepoint), ^b^exact McNemar – test, ^c^Mann Whitney U – test, ^d^Wilcoxon signed rank – test, ^e^Effect size (Cohen’s d): (first visit mean – follow up mean)/ SD (first visit). ^f^ Effect size (Cohen’s d): for two independent samples using groups (JIA/Controls)

*OHRQoL* oral health related quality of life, *ADD scores* additive scores, *ECOHIS* early childhood oral health impaction scale, *Child OIDP* child oral impact on daily performances, *JIA* juvenile idiopathic arthritis, *n/N*  number observed/total number assessed, *SD* standard deviation, *CI* confidence interval. *P*-values < 0.05 are marked in bold

The frequencies for Child OIDP > 0 and the mean ADD scores are presented in Table 3. More adolescents with JIA reported negative impacts on daily performance at the first visit than at the two-year follow-up (27.0% and 15.3%, respectively, *p* = *0.004*). Stratified by gender, we found that girls with JIA more commonly reported Child OIDP > 0 than boys with JIA (42% and 9% respectively, *p* < *0.001*) and girls without JIA *(*21%*, p* = *0.018*) at the first visit but not at the two-year follow-up (results not shown). Adolescent girls with JIA had a higher mean Child OIDP ADD score than adolescent boys with JIA (first visit: 1.76 and 0.46, respectively, *p* < *0.001,* two-year follow-up: 0.81 and 0.21, *p* = *0.032*) and girls without JIA at the first visit (first visit: 0.52, *p* = *0.008,* two-year follow-up: 0.38, *p* = *0.444*). We did not find this difference when comparing boys with and without JIA. The mean Child OIDP ADD scores for both the JIA and control group were significantly lower at the two-year follow-up, with a low effect size (Table 3). When analyzing and comparing each separate item, *problems with eating* were reported by more adolescents with JIA than by adolescents in the control group (19.8% and 9.8% respectively, *p* = *0.041*) at the first visit (results not shown). There was no significant difference between adolescents in the JIA and the control group according to any other item at either of the study visits.

### Internal consistency and validity of the ECOHIS and Child OIDP

The total ECOHIS scale showed good internal consistency for the JIA group (Cronbach’s alpha = 0.82 at both visits) and acceptable internal consistency for the control group (Cronbach’s alpha = 0.77 at the first visit and 0.74 at the two-year follow-up) (Supplemental, Additional file S3, Table 1). At both visits we found lower mean ECOHIS ADD scores for participants with ratings of “good” oral health and satisfaction with tooth appearance, and correspondingly higher scores when ratings of oral health were poor and there was dissatisfaction with tooth appearance (Table 4). The ECOHIS change ADD scores were small in both the JIA and control group and only correlated with changes in the global oral health outcomes for children with JIA (Supplemental, Additional file S3, Table 2)*.*

**Table 4:** Mean OHRQoL ADD scores by category for global reference variable

Reference variable	Good/satisfied				Poor/unsatisfied^b^				
	N	Mean	(SD)	[95% CI]	N	Mean	(SD)	[95% CI]	*p–*value^*a*^
Children—ECOHIS									
Oral health—First visit									
JIA	40	2.98	(0.53)	[1.91–4.04]	5	11.00	(1.50)	[7.98–14.02]	** < *0.001***
Control	53	2.08	(0.37)	[1.33–2.82]	1	1	(Only one observation)		***-***
Tooth appearance—First visit									
JIA	36	2.72	(0.59)	[1.54–3.90]	9	8.44	(1.17)	[6.08–10.81]	***0.008***
Control	50	1.76	(0.35)	[1.06–2.46]	4	5.75	(1.24)	[3.26–8.24]	*0.104*
Oral health—Two-Year Follow-up									
JIA	34	3.68	(0.76)	[2.15–5.21]	11	7.82	(1.34)	[5.13–10.51]	***0.019***
Control	52	2.48	(0.43)	[1.63–3.34]	2	5.50	(2.17)	[1.14–9.86]	*0.203*
Tooth appearance—Two-Year Follow-up									
JIA	35	3.40	(0.69)	[2.00–4.80]	10	9.20	(1.30)	[6.58–11.82]	***0.001***
Control	47	2.38	(0.45)	[1.48–3.28]	7	4.00	(1.16)	[1.66–6.34]	***0.029***
Adolescents – Child OIDP									
Oral health—First visit									
JIA	86	1.15	(2.64)	[0.58–1.72]	24	1.21	(2.25)	[0.26–2.16]	*0.967*
Control	87	0.45	(1.26)	[0.18–0.72]	15	0.87	(1.85)	[-0.16–1.89]	*0.211*
Tooth appearance—First visit									
JIA	79	0.89	(2.08)	[0.42–1.35]	31	1.74	(3.41)	[0.49–2.99]	*0.088*
Control	75	0.39	(1.27)	[0.09–0.68]	27	0.85	(1.56)	[0.23–1.47]	***0.021***
Oral health—Two-Year Follow-up									
JIA	99	0.32	(1.13)	[0.10–0.55]	12	2.25	(3.14)	[0.26–4.24]	***0.001***
Control	96	0.22	(0.81)	[0.05–0.38]	6	0.83	(0.75)	[0.04–1.62]	***0.006***
Tooth appearance—Two-Year Follow-up									
JIA	95	0.34	(1.15)	[0.10–0.57]	16	1.69	(2.87)	[0.16–3.22]	***0.010***
Controls	80	0.20	(0.82)	[0.02–0.38]	22	0.45	(0.80)	[0.10–0.81]	***0.020***

^a^Mann-Whitney U exact probability. ^b^including neither/nor

*OHRQoL* oral health related quality of life, *ADD* additive, *SD* standard deviation, *CI* confidence interval, *ECOHIS* early childhood oral health impaction scale, *JIA* juvenile idiopathic arthritis, *Child OIDP* child oral impact on daily performances. *P*-values < 0.05 are marked in bold

The internal consistency reliability (Cronbach’s alpha) of the Child OIDP inventory was 0.77 for the JIA group and 0.73 for the control group at the first visit and 0.69 and 0.65, respectively, at follow-up (Supplemental, Additional file S3, Table 1). The first visit and two-year follow-up mean Child OIDP ADD scores were lower for those rating oral health as good and were satisfied with their tooth appearance, while the scores were higher for those rating oral health as poor or were unsatisfied with their tooth appearance (Table 4). The Child OIDP change ADD scores were small and did not correlate with changes in the patient-rated overall oral health and tooth appearance (Supplemental, additional file S3, Table 2).

### Associations between OHRQoL and orofacial pain, and impaired physical health,

Significantly more children and adolescents with JIA reported orofacial pain than controls at both visits (Tables 1 and 2).In the group of children under 12 years of age, all children who reported orofacial pain also reported at least one negative impact on OHRQoL at follow-up (Table 5). Independent of group affiliation, adolescents with orofacial pain were more likely to report negative OHRQoL impacts than those without orofacial pain (Table 6). Children and adolescents reporting orofacial pain had higher mean OHRQoL ADD scores (Supplemental, additional file S4, Table 1). These results were significant for children with and without JIA at the two-year follow-up and for adolescents with JIA both at the first visit and at the two-year follow-up.

**Table 5:** Orofacial pain, impaired CHQ, disease activity, and TMJ-arthritis in relation to OHRQoL impacts in children

	**First visit ECOHIS > 0**							**Two-year follow-up ECOHIS > 0**						
	**Unadjusted regressions**				**Adjusted regressions**^**a**^			**Unadjusted regressions**				**Adjusted regressions**^**a**^		
	n	OR	95%CI	*p-value*	OR	95%CI	*p-value*	n	OR	95%CI	*p-value*	OR	95%CI	*p-value*
All children (with and without JIA)														
Orofacial pain – EVER^b^														
No	82	Ref			Ref			83	Ref					
Yes	19	3.9	0.8–18.4	*0.080*	3.1	0.7–15.1	*0.155*	18	*			*		
CHQ physical score impaired														
No	87	Ref			Ref			82	Ref					
Yes	9	3.2	0.4–27.1	*0.281*	1.9	0.2–18.0	*0.575*	14	4.2	0.5–34.1	*0.180*	3.6	0.4–31.1	*0.247*
Children with JIA only														
JADAS 71 > 0														
No	13	Ref			Ref			17	Ref					
Yes	33	2.5	0.5–11.3	*0.238*	2.8	0.5–15.7	*0.254*	29	1.3	0.3–6.9	*0.726*	1.5	0.3–7.9	*0.665*
TMJ involvement – EVER^b^														
No	36	Ref			Ref			34	Ref					
Yes	11	*			*			13	2.6	0.3–23.7	*0.405*	4.0	0.4–42.2	*0.249*

Unadjusted and adjusted ordinal logistic regressions including both children with and without JIA for orofacial pain and CHQ physical score

^a^adjusted for group affiliation (JIA/Controls) when both children with and without JIA were included, gender and educational level of mother and father. ^b^due to the missing timeframe of ECOHIS the occurrence of orofacial pain –ever, and TMJ involvement –ever (as recorded clinically by the pediatric rheumatologist) was used in these analyses for children

*Informative model results not available (N/A) from the model because of a perfect correlation, all children with orofacial pain/TMJ arthritis have ECOHIS > 0

*CHQ* child health questionnaire, *TMJ* temporomandibular joint, *OHRQoL* oral health related quality of life, *ECOHIS* early childhood oral health impaction scale, *n* number, *OR* odds ratio, *CI* confidence interval, *JIA* juvenile idiopathic arthritis, *JADAS* juvenile arthritis disease activity score. *P*-values < 0.05 are marked in bold

There were no significant associations between negative OHRQoL impacts and impaired physical health in children with JIA. Adolescents with JIA and impaired physical health had significantly higher odds of having at least one negative OHRQoL impact at the two-year follow-up (adjusted logistic regression, OR 5.1, 95% CI 1.2–20.8), as shown in Table 6. The mean Child OIDP ADD scores for adolescents with JIA and impaired physical health were higher both at the first visit and the two-year follow-up (Supplemental, additional file S4, Table 2).

**Table 6:** Orofacial pain, impaired CHQ, disease activity, and TMJ-arthritis in relation to OHRQoL impacts in adolescents

	**First visit Child OIDP > 0**							**Two-year follow-up Child OIDP > 0**						
	**Unadjusted regressions**				**Adjusted regressions**^**a**^			**Unadjusted regressions**				**Adjusted regressions**^**a**^		
	n	OR	95%CI	*p-value*	OR	95%CI	*p-value*	n	OR	95%CI	*p-value*	OR	95%CI	*p-value*
*Adolescents with and without JIA*														
Orofacial pain the last 30 days^b^														
No	159	Ref			Ref			162	Ref			Ref		
Yes	33	4.2	1.9–9.3	** < *0.001***	4.0	1.6–10.0	***0.003***	30	5.3	2.1–13.1	** < *0.001***	5.3	1.9–14.9	***0.001***
CHQ physical score impaired														
No	136	Ref			Ref			130	Ref			Ref		
Yes	21	2.6	1.0–6.9	*0.055*	3.0	1.0–9.4	*0.055*	27	3.4	1.2–9.8	***0.021***	5.1	1.2–20.8	***0.024***
*Adolescents with JIA*														
JADAS 71 > 0														
No	36	Ref			Ref			37	Ref			Ref		
Yes	61	5.8	1.6–21.1	***0.008***	5.1	1.3–19.6	***0.017***	60	2.3	0.6–8.8	*0.239*	1.8	0.4–7.6	*0.418*
TMJ involvement at study visit^b^														
No	93	Ref			Ref			98	Ref			Ref		
Yes	12	12.5	3.1–50.9	** < *0.001***	8.5	2.0–37.0	***0.004***	7	2.6	0.5–14.9	*0.279*	1.8	0.3–11.1	*0.526*

Unadjusted and adjusted ordinal logistic regressions including both adolescents with and without JIA for orofacial pain and CHQ physical score

^a^adjusted for group affiliation (JIA/Controls) when both adolescents with and without JIA were included, gender and educational level of mother and father. ^b^due to the timeframe of Child OIDP (last three month) the occurrence of orofacial pain – last 30 days and TMJ involvement – at study visit (as recorded clinical by the pediatric rheumatologist) was used in these analyses for adolescents

*CHQ* child health questionnaire, *TMJ* temporomandibular joint, *OHRQoL* oral health related quality of life, *Child OIDP* child oral impact on daily performance, *N* number, *OR* odds ratio, *CI* confidence interval, *JIA* juvenile idiopathic arthritis, *JADAS* juvenile arthritis disease activity score. *P*-values < 0.05 are marked in bold

### Associations between OHRQoL and JIA disease activity and TMJ involvement

The mean OHRQoL ADD scores were higher for children and adolescents with active disease (JADAS71 ≥ 1). The differences in ECOHIS and Child OIDP add scores according to disease activity were significant only at the first visit (Supplemental, additional file S4, Table 3). Among adolescents, we found significantly higher odds for negative OHRQoL impacts in individuals with active disease (OR 5.1, 95% CI 1.3–19.6) at the first visit, as presented in Table 6.

All children with JIA and clinical TMJ involvement had at least one negative impact on OHRQoL (ECOHIS > 0) at the first visit (Table 5). Adolescents with JIA and TMJ involvement had significantly higher odds of reporting negative impacts on OHRQoL at the first visit (OR 8.5 95% CI 2.0–37.0), but this association was not found at the two-year follow-up (Table 6).

## Discussion

In this longitudinal multicenter study focusing on children and adolescents with JIA, we found that there are differences in oral health and OHRQoL between participants with and without JIA. Children with JIA reported pain or discomfort during toothbrushing and ulcerations more often than controls. Orofacial pain was also most frequent in children and adolescents with JIA. Furthermore, moderate to poor oral hygiene and gingival bleeding were more common among adolescents with JIA than among those without JIA. Children with JIA had higher OHRQoL mean ADD scores than children without JIA. There were no significant differences in frequencies of negative OHRQoL impacts or in OHRQoL mean ADD scores in adolescents with and without JIA. Testing for longitudinal validity, the OHRQoL change ADD scores were small in both the JIA and control group, and correlated only with changes in the global oral health outcomes for children with JIA. We found associations between negative OHRQoL impacts and orofacial pain, impaired physical health, active JIA disease, and TMJ involvement.

A strength of this study is the longitudinal study design and the included age- and gender-matched control group. It is difficult, to define the right interval for a longitudinal study on children and adolescents with such a wide age span, since changes in children can appear quickly in certain time periods of cognitive development and growth. Rahimi et al. [13] reported persistency of orofacial symptoms in a two-year follow-up of adolescents with JIA, and we found this interval sufficient for the NorJIA study. An additional strength of the study is the use of standardized and validated protocols and age-appropriate questionnaires. Furthermore, we conducted calibration exercises among the dental examiners both before and during the study period. Another strength of the NorJIA study is the multidisciplinary collaboration of the medical and dental specialists, that increases the knowledge beyond the individual specialists’ fields and hopefully lead to a more comprehensive understanding of the disease and a better treatment.

A limitation of this study is the small number of participants in the group of children < 12 years. Approximately half of the children could not be included since they changed age group and reported OHRQoL in the Child OIDP instead of the ECOHIS at the two-year follow-up. Hence, the sample size is low for some analyses for subgroups of our study population. An additional limitation regarding the ECOHIS questionnaire is the different timing of completion; some parents completed the questionnaire after the examination. Another limitation of this report is the assessment of TMJ involvement, that was based on the best judgment of the pediatric rheumatologist without MRI scoring available.

To our knowledge, NorJIA is the first study that applied the ECOHIS and the Child OIDP inventory in a follow-up study of children and adolescents with JIA. While most of the children reported negative impacts on OHRQoL (ECOHIS > 0), less than one-third of adolescents reported oral impacts on daily life activities (Child OIDP > 0). A higher frequency of negative OHRQoL impacts in children than in adolescents was also found in a previous Norwegian study, where ECOHIS and Child OIDP inventories were used in a general local population of children and adolescents [29]. Importantly, the ECOHIS and Child OIDP instruments are not directly comparable since the Child OIDP addresses impacts experienced during the last three months, while ECOHIS items are reported with no time limit. To be able to compare our results with prior publications, we chose to use the same methods of dichotomization of the ECOHIS instrument as previous Norwegian studies. Thus, the severity of the impacts on the child’s quality of life can be discussed if the impact occurred “hardly ever”. This must be taken into account, when evaluating the high number of children with impacts.

We found no differences in the number of participants that reported negative impacts on OHRQoL in the JIA group compared to controls, but we found a significantly higher mean ECOHIS ADD score for children with JIA with a moderate effect size at both visits. These findings may indicate, that children with JIA have several negative impacts or have impacts more often than children without JIA. In contrast, Santos et al. [12] reported no difference in mean OHRQoL scores in a study on 17 children and adolescents with JIA and 15 controls using the short form of the Brazilian Parental-Caregiver Perception Questionnaire. While we found, that the OHRQoL in children was stable over time, fewer adolescents had negative impacts on daily performance at the two-year follow-up compared to the first visit. In a review, Alvarez et al. [42] found lower Child OIDP scores in studies carried out in groups of older adolescents compared to studies with younger cohorts. The lower frequency of reported negative impacts in adolescents at the two-year follow-up in our study may thus be related to increased age. There were no significant changes in oral health in terms of other reported orofacial symptoms or clinical findings such as caries or gingival bleeding. However continuous medical treatment and multidisciplinary follow up may have contributed to less negative OHRQoL impacts.

The reliability in terms of internal consistency for the total ECOHIS scale was good at the first visit and acceptable at the two-year follow-up. For the Child OIDP scale, the internal consistency was acceptable at the first visit but somewhat lower than the accepted range for the two-year follow-up [43]. The Child OIDP inventory has only eight items. The low number of items in a scale can affect Cronbach’s alpha and lead to an underestimation of the reliability in short scales [43]. A recent reliability generalization meta-analysis of the Child OIDP questionnaire showed that a majority of publications reported a Cronbach’s alpha of ≥ 0.7, and they found that age and gender have a significant effect on the overall estimate [44].

Testing for criterion validity, we found higher mean ECOHIS and Child OIDP ADD scores in relation to the global reference outcomes “poor oral health” and “unsatisfied with tooth appearance”, indicating that both OHRQoL instruments are valid at both visits. Assessing the longitudinal validity of the ECOHIS and Child OIDP in our study cohort, most of the children and adolescents were stable in their category for the global oral health outcomes, and the mean change ADD scores for ECOHIS and Child OIDP were low. Only a few participants had a worsened or improved global rating of oral health and tooth appearance, and in both groups were as well participants that had negative and positive change scores. The same problem was described by Locker et al., testing the responsiveness of the oral health impact profile [32]. Intervention studies on early childhood caries that applied the ECOHIS instrument found mild to moderate changes in the ADD scores after treatment [30, 31, 45]. As the present NorJIA study is an observational study without major interventions on oral health, significant changes were not expected. Mashoto et al. [46] used the Child OIDP instrument in a study on Tanzanian children that underwent caries treatment. They described flooring effects where 63% of the total group and 50% of the treated participants had a zero score at baseline [46]. In our study, the frequency of adolescents without negative oral impacts on daily activities was even higher with approximately 70% of the adolescents with JIA and 80% of controls. This flooring effect may additionally limit the sensitivity of the Child OIDP to changes in the global oral health categories.

We found higher OHRQoL ADD scores for children and adolescents with orofacial pain, impaired physical health, or active JIA disease. Leksell et al. concluded in a study of 41 patients with JIA and 41 controls that symptoms from the orofacial area were more common in the JIA group and impacted daily life to a high extent [14]. Comparable results were found in a more recent two-year prospective observational study of 157 Danish children with JIA. Rahimi et al. applied the Child Perceptions Questionnaire (a 31-item questionnaire on OHRQoL) at the two-year follow-up and found that orofacial pain and functional disability significantly reduced OHRQoL [13]. Although these studies are not directly comparable due to different study designs, they support our results that orofacial pain correlates with increasing negative impacts on OHRQoL in children and adolescents.

Adolescents with JIA and impaired physical health had significantly more negative impacts on OHRQoL than those with normal physical health. Two cross-sectional studies on associations between OHRQoL and general HRQoL in mainly adult populations indicate that OHRQoL has some impact on HRQoL and that low HRQoL can be an indicator of impaired oral health [47, 48]. We found no comparable studies of associations between OHRQoL and HRQoL in children and adolescents. Analyzing the whole first-visit JIA cohort of the NorJIA study (JIA, *n* = 221), Gil et al. reported a higher risk for negative impacts on daily activities in adolescents with JIA and continued disease activity or flare [49]. In our report which included 111 of the 125 adolescents with JIA and 47 of the 96 children with JIA, we analyzed the associations between the OHRQoL ADD scores and the JADAS71 score, a composite measure for disease activity. We found similar associations as Gil et al. with significantly higher mean ECOHIS and Child OIDP scores in the participants with active disease (JADAS ≥ 1) at the first visit. However, the mean OHRQoL scores for participants with and without active disease did not differ significantly at the two-year follow-up.

At the first visit children and adolescents with JIA and TMJ involvement had higher odds for negative OHRQoL impacts than those without TMJ involvement. Clinical findings as active TMJ arthritis orofacial pain or dysfunction were treated according to the clinical protocols between first visit and two-year follow-up. The treatment could involve adjustment of the systemic medication in cases of active TMJ arthritis, information and physical exercises or splint therapy in cases of orofacial pain or dysfunction. The individually tailored multidisciplinary treatment may explain, that we did not find significantly higher odds for negative OHRQoL impacts in participants with TMJ involvement and JADAS > 1 at the two year follow up (24).

## Conclusions

Our cohort study found differences in the oral health and OHRQoL of children and adolescents with JIA compared to controls. Children and adolescents with orofacial pain were more likely to report negative oral health-related impacts on daily life activities than those without pain. We found an association between impaired OHRQoL and active disease (JADAS ≥ 1), impaired physical health, and TMJ involvement. Adolescent girls with JIA reported negative OHRQoL impacts more often than both boys with JIA and girls without JIA. Future research should identify and explore OHRQoL instruments that may better capture the OHRQoL impacts in children and adolescents with JIA. Pediatric rheumatologists and dental specialists should give attention to children and adolescents reporting impaired physical health, high disease activity, orofacial pain, and TMJ involvement in JIA and cooperate closely to optimize normal daily life activities and functioning.

### Supplementary Information

**Additional file 1: **Original codes and recoded variables. **S1 Table 1.** Categories for socio-behavioral characteristics, as originally coded and as recoded for analyses. **S1 Table 2**. ECOHIS answering categories as originally coded and recoded for analysis.

**Additional file 2: **Dropout analysis. **S2 Table 1**. First visit characteristics for children <12 years in JIA and control group for dropout analysis. **S2 Table 2.** First visit characteristics for adolescents ≥12 years in JIA and control group for dropout analysis.

**Additional file 3: **Reliability and validity of OHRQoL instruments. **S3 Table 1.** Internal consistency reliability of the OHRQoL instruments. **S3 Table 2. **Mean OHRQoL change ADD scores by change categories of reference variables in children and adolescents.

**Additional file 4: **Mean OHRQoL ADD scores according to orofacial pain, CHQ physical scores, and disease activity (JADAS71). **S4 Table 1.** Mean OHRQoL ADD scores according to orofacial pain. **S4 Table 2.** Mean OHRQoL ADD scores according to CHQ physical scores. **S4 Table 3.** Mean OHRQoL ADD scores according to JADAS71.

## Data Availability

The datasets used and/or analyzed during the current study are available from the corresponding author upon reasonable request.

## Abbreviations

ADD: Additive
Child-OIDP: Child oral impact on daily performance
CHQ-PF50: 50 Items parent administered version of the child health questionnaire
CI: Confidence interval
CRP: C-reactive protein
d_1-5_f/ D_1-5_F: Caries on enamel and dentin level in primary/permanent teeth
ECOHIS: Early childhood oral health impact scale
ES: Effect size
ESR: Erythrocyte sedimentation rate
GBI: Gingival Bleeding index
HRQoL: Health related quality of life
ILAR: The International League of Associations for Rheumatology
JADAS: Juvenile arthritis disease activity score
JIA: Juvenile idiopathic arthritis
MDgloVAS: The pediatric rheumatologist’s global assessment of disease activity
NorJIA: Norwegian JIA Study
OHI-S: Simplified Oral Hygiene Index
OHRQoL: Oral health related quality of life
OR: Odds ratio
PhS: Physical score of the CHQ-PF50
PRgloVAS: Patients/parents global rating of the diseases influence on wellbeing
SD: Standard deviation
TMJ: Temporomandibular joint
VAS: Visual analog scale
