# Supplementary material for: Oral health-related quality of life, impaired physical health and orofacial pain in children and adolescents with juvenile idiopathic arthritis – a prospective multicenter cohort study
Source: BMC Oral Health. 2023 Nov 20;23:895. doi: 10.1186/s12903-023-03510-0 (PMC10662257; doi:10.1186/s12903-023-03510-0)
Supplement: Supplementary file 1 — Additional file 1: Original codes and recoded variables. S1 Table 1. Categories for socio-behavioral characteristics, as originally coded and as recoded for analyses. S1 Table 2. ECOHIS answering categories as originally coded and recoded for analysis. [file 12903_2023_3510_MOESM1_ESM.pdf]

Supplementary information

Additional file S1 – original codes and recoded variables

S1 Table 1. Categories for socio-behavioral characteristics, as originally coded and as recoded for analyses.

Variable	Categories	Original code	New code
Share household with	Mother and father	1	0
	Only mother	2	1
	Only father	3	1
	Other, specify	4	0/1*
Educational level of mother/father	Primary school	1	1
	High school/vocational school	2	1
	University/college (≤4 years)	3	0
	University/college (≥5 years)	4	0
	Unknown	90	[missing]
	Missing	99	[missing]
Frequency of toothbrushing	Never	1	1
	Most days	2	1
	Once a day	3	1
	Twice a day, or more	4	0
	Do not know	90	1
	Missing	99	[missing]
Frequency of tooth flossing during the last 3 months	Several times daily	1	0
	Twice a day	2	0
	Daily	3	0
	Several times weekly	4	0
	Several times a month, but not weekly	5	1
	Seldom	6	1
	Never	7	1
	Do not know	90	1
	Missing	99	[missing]
Gingival bleeding occurs during toothbrushing	Every day	1	1
	Most days	2	1
	Once a week	3	1
	Sometimes	4	1
	Never	5	0
	Do not know	90	0
	Missing	99	[missing]
Pain or discomfort occurs during toothbrushing (adolescents ≥12 years)	Yes	1	1
	No	2	0
	Do not know	90	0
Merged with:	Missing	99	[missing]
Impression of child's experience during toothbrushing (children <12 years)	Painful	1	1
	Unpleasant	2	1
	Okay	3	0
	Do not know	90	0
	Missing	99	[missing]
Frequency of intraoral ulcerations	Once or several times a month	1	1
	Several times yearly, but less than once a month	2	1
	Less than once a year	3	0
	Never	4	0
	Do not know	90	0
	Missing	99	[missing]

*living across two households, given two caregivers in both households coded 1.

S1 Table 2. ECOHIS answering categories as originally coded and recoded for analysis

Category	Original code	New code for ADD score
Never	1	0
Hardly ever	2	1
Occasionally	3	2
Often	4	3
Very often	5	4
Do not know	90	0-4*
Missing	99	0-4*

* Replaced by the average score for section if less than three missing items for the child section or less than two missing items for the family section; participants with more than two items missing from the child section or more than one item missing from the family section have been excluded (n=1).
