# Supplementary material for: Oral health-related quality of life, impaired physical health and orofacial pain in children and adolescents with juvenile idiopathic arthritis – a prospective multicenter cohort study
Source: BMC Oral Health. 2023 Nov 20;23:895. doi: 10.1186/s12903-023-03510-0 (PMC10662257; doi:10.1186/s12903-023-03510-0)
Supplement: Supplementary file 2 — Additional file 2: Dropout analysis. S2 Table 1. First visit characteristics for children <12 years in JIA and control group for dropout analysis. S2 Table 2. First visit characteristics for adolescents ≥12 years in JIA and control group for dropout analysis. [file 12903_2023_3510_MOESM2_ESM.pdf]

Additional file S2 – Dropout analysis

S2 Table 1. First visit characteristics for children <12 years in JIA and control group for dropout analysis

Variable	JIA					Controls				
	Followed up		Lost to Follow-up			Followed up		Lost to Follow-up		
	%	(n/N)	%	(n/N)	*p-value*	%	(n/N)	%	(n/N)	*p-value*
Median age, years (IQR)	7.6	(6.7-8.9)	10.7	(9.9-11.5)	***<0.001***[a]	7.8	(6.8-9.1) *	10.6	(9.8-11.3)	***<0.001***[a]
Female gender	70.2	(33/47)	65.3	(32/49)	*0.607*[b]	75.9	(41/54)	61.4	(27/44)	*0.120*[b]
Education level of Mother, university/college	63.8	(30/47)	75.5	(37/47)	*0.213*[b]	61.1	(33/54)	72.7	(32/44)	*0.226*[b]
Education level of Father, university/college	36.2	(17/47)	49.0	(24/49)	*0.205*[b]	53.7	(29/54)	56.8	(25/44)	*0.758*[b]
Share household with two caregivers	85.1	(40/47)	83.7	(41/49)	*0.847*[b]	92.6	(50/54)	95.5	(42/44)	*0.688*[b]
Toothbrushing, twice a day or more	68.1	(32/47)	75.5	(37/49)	*0.419*[b]	75.9	(41/54)	81.8	(36/44)	*0.480*[b]
Tooth flossing, several times a week/more	10.6	(5/47)	22.5	(11/49)	*0.171*[b]	11.3	(6/52)	13.6	(6/44)	*0.730*[b]
Gingival bleeding during toothbrushing, sometimes or more	43.5	(20/46)	47.9	(23/48)	*0.666*[b]	33.3	(18/54)	36.4	(16/44)	*0.754*[b]
Pain or discomfort during toothbrushing	6.4	(3/47)	6.3	(3/48)	*1.000*[b]	1.9	(1/54)	2.3	(1/44)	*1.000*[b]
Ulcerations, several times yearly or more	31.1	(14/45)	18.8	(9/48)	*0.167*[b]	13.0	(7/54)	20.5	(9/44)	*0.318*[b]
Orofacial pain ever	27.7	(13/47)	42.9	(21/49)	*0.120*[b]	11.1	(6/54)	6.8	(3/44)	*0.509*[b]
Orofacial pain the last 30 days	14.9	(7/47)	18.4	(9/49)	*0.648*[b]	1.9	(1/54)	2.3	(1/44)	*1.000*[b]
Caries, $d_{1\text{-}5}f/D_{1\text{-}5}F$*-level >0*	26.1	(12/46)	35.4	(17/48)	*0.328*[b]	27.8	(15/54)	34.1	(15/44)	*0.500*[b]
ECOHIS >0	80.9	(38/47)	79.6	(39/49)	*0.877*[b]	64.8	(35/54)	72.7	(32/44)	*0.402*[b]
Poor dental health[c]	11.1	(5/45)	17.0	(8/47)	*0.552*[b]	1.9	(1/54)	4.6	(2/44)	*0.586*[b]
Not satisfied with tooth appearance[c]	20.0	(9/45)	31.9	(15/47)	*0.193*[b]	7.4	(4/54)	11.4	(5/44)	*0.727*[b]
Impaired physical health (CHQ PhS <40)	19.6	(9/46)	31.3	(15/48)	*0.194*[b]	0	(0/52)	2.5	(1/40)	*0.435*[b]

[a]Mann-Whitney U -test. [b]Chi squared- /Fisher's exact-test. [c]including neither/nor. JIA =juvenile idiopathic arthritis. IQR =interquartile range. n/N =number observed/total number assessed for each variable, excluding missing values. $d_{1\text{-}5}f/D_{1\text{-}5}F$ =decayed and filled deciduous/ permanent teeth, enamel caries included. ECOHIS =early childhood oral health impaction scale. CHQ =child health questionnaire. PhS =physical summary score. P-values <0.05 are marked in bold.

S2 Table 2. First visit characteristics for adolescents ≥12 years in JIA and control group for dropout analysis

Variable	JIA					Controls				
	Followed up		Lost to Follow-up			Followed up		Lost to Follow-up		
	%	(n/N)	%	(n/N)	*P - value*	%	(n/N)	%	(n/N)	*P value*
Median age, years (IQR)	14.4	(13.3-15.3)	15.5	(14.9-15.8)	***0.015*** [a]	14.7	(13.3-15.5)	14.9	(13.8-15.8)	*0.170* [a]
Female gender	53.2	(59/111)	50.0	(7/14)	*0.824* [b]	54.9	(56/102)	36.4	(8/22)	*0.115* [b]
Education level of mother, university/college	60.8	(65/107)	38.5	(5/13)	*0.124* [b]	73.1	(68/93)	90.0	(18/20)	*0.108* [b]
Education level of father, university/college	39.6	(42/106)	15.4	(2/13)	*0.128* [b]	63.2	(60/95)	57.9	(11/19)	*0.611* [b]
Share household with two caregivers	77.5	(79/102)	84.6	(11/13)	*0.731* [b]	76.5	(78/102)	85.0	(17/20)	*0.560* [b]
Toothbrushing, twice a day or more	79.1	(83/105)	84.6	(11/13)	*1.000* [b]	75.5	(77/102)	85.0	(17/20)	*0.561* [b]
Tooth flossing, several times a week or more	22.1	(23/104)	15.4	(2/13)	*0.732* [b]	22.6	(23/102)	25.0	(5/20)	*0.777* [b]
Gingival bleeding during toothbrushing, sometimes or more	61.5	(64/104)	46.2	(6/13)	*0.286* [b]	58.8	(60/102)	40.0	(8/20)	*0.121* [b]
Pain or discomfort during toothbrushing	10.7	(11/103)	0	(0/13)	*0.216* [b]	13.7	(14/102)	10.5	(2/19)	*1.000* [b]
Ulcerations, several times yearly or more	25.0	(25/100)	23.1	(3/13)	*1.000* [b]	36.3	(37/102)	45.0	(9/20)	*0.462* [b]
Orofacial pain ever	42.7	(47/110)	61.5	(8/13)	*0.244* [b]	14.7	(15/102)	27.3	(6/22)	*0.154* [b]
Orofacial pain the last 30 days	28.2	(31/110)	46.2	(6/13)	*0.181* [b]	2.9	(3/102)	13.6	(3/22)	*0.068* [b]
Caries - $D_{1\text{-}5}$F-level >0	52.3	(56/107)	85.7	(12/14)	***0.018*** [b]	57.8	(59/102)	40.9	(9/22)	*0.148* [b]
Child OIDP >0	27.0	(30/111)	21.4	(2/14)	*0.466* [b]	20.6	(21/102)	27.3	(6/22)	*0.491* [b]
Poor dental health[c]	21.8	(24/110)	21.4	(3/14)	*1.000* [b]	14.7	(15/102)	9.1	(2/22)	*0.735* [b]
Not satisfied with tooth appearance[c]	28.2	(31/110)	42.9	(6/14)	*0.258* [b]	26.5	(27/102)	36.4	(8/22)	*0.350* [b]
Impaired physical health (CHQ PhS <40)	22.6	(24/106)	28.6	(4/14)	*0.737* [b]	0	(0/92)	0	(0/22)	-

[a]Mann Whitney U-test. [b]Chi squared- /Fisher's exact-test. [c]including neither/nor. JIA =juvenile idiopathic arthritis. IQR =interquartile range. n/N =number observed/total number assessed for each variable, excluding missing values. $D_{1\text{-}5}F$ =decayed and filled permanent teeth, enamel caries included. Child OIDP =child oral impact on daily performances. CHQ =child health questionnaire. PhS =physical summary score. P-values <0.05 are marked in bold.
