# Supplementary material for: Oral health-related quality of life, impaired physical health and orofacial pain in children and adolescents with juvenile idiopathic arthritis – a prospective multicenter cohort study
Source: BMC Oral Health. 2023 Nov 20;23:895. doi: 10.1186/s12903-023-03510-0 (PMC10662257; doi:10.1186/s12903-023-03510-0)
Supplement: Supplementary file 3 — Additional file 3: Reliability and validity of OHRQoL instruments. S3 Table 1. Internal consistency reliability of the OHRQoL instruments. S3 Table 2. Mean OHRQoL change ADD scores by change categories of reference variables in children and adolescents. [file 12903_2023_3510_MOESM3_ESM.pdf]

Additional file S3 – Reliability and validity of OHRQoL instruments

S3 Table 1. Internal consistency reliability of the OHRQoL instruments

	**Number of items**	**Cronbach's alpha**	**Average interitem covariance**
ECOHIS			
Total scale - First visit	13	0.81	0.06
JIA - First visit	12	0.82	0.10
Controls - First visit	13	0.77	0.03
Total scale - Follow-up	13	0.79	0.08
JIA - Follow-up	13	0.82	0.11
Controls - Follow-up	12	0.74	0.05
Child OIDP			
Total scale - First visit	8	0.76	0.05
JIA - First visit	8	0.77	0.08
Controls - First visit	8	0.73	0.02
Total scale - Follow-up	8	0.68	0.02
JIA - Follow-up	8	0.69	0.03
Controls - Follow-up	7	0.65	0.01

ECOHIS =early childhood oral health impaction scale. JIA =juvenile idiopathic arthritis. OIDP =oral impact on daily performances. JIA =juvenile idiopathic arthritis.

S3 Table 2. Mean OHRQoL change ADD scores by change categories of reference variables in children and adolescents

	Change in the category of the reference variable						
	**Worsened**		**Stable**		**Improved**		**Total**
	n	Mean (SD)	n	Mean (SD)	n	Mean (SD)	Mean (SD)[95%CI]
Satisfaction with oral health							
Children - ECOHIS change ADD score							
JIA	7	-2.6 (2.8)	35	-0.4 (4.0)	1	6 (0)	-1.0 (4.0) [-2.1-0.2]
Controls	2	-2.0 (0)	51	-0.5 (3.4)	1	-1 (0)	- 0.5 (3.3) [-1.4-0.4]
Adolescents - Child OIDP change ADD score							
JIA	4	-0.8 (1.5)	90	0.7 (2.7)	16	0.7 (2.0)	0.6 (2.6) [0.1-1.1]
Controls	2	2.0 (2.8)	89	0.2 (1.3)	11	0.3 (0.8)	0.3 (1.3) [0.0-0.5]
Satisfaction with tooth appearance							
Children - ECOHIS change ADD score							
JIA	5	-3.6 (3.2)	34	-0.6 (3.7)	4	3.5 (3.8)*	
Controls	5	-2.2 (1.5)	47	-0.3 (3.4)	2	-2.5 (0.7)	
Adolescents - Child OIDP change ADD score							
JIA	4	0.25 (0.5)	87	0.4 (2.1)	19	1.5 (4.2)	
Controls	6	-0.5 (1.2)	85	0.3 (1.3)	11	0.1 (1.1)	
Gingival bleeding during toothbrushing							
Children - ECOHIS change ADD score							
JIA	7	-0.7 (2.3)	34	-1.2 (4.3)	4	-0.5 (5.2)	
Controls	9	-1.3 (3)	34	-0.6 (3.3)	8	0.6 (3.7)	
Adolescents - Child OIDP change ADD score							
JIA	14	-0.3 (1.6)	69	0.5 (2.0)	15	1.5 (3.7)	
Controls	20	0.7 (1.9)	65	0.2 (1.1)	16	0.1 (0.6)	
Pain or discomfort during toothbrushing							
Children - ECOHIS change ADD score							
JIA	1	-2 (0)	45	-1.0 (4.1)	0		
Controls	0		50	-0.6 (3.3)	1	1 (0)	
Adolescents - Child OIDP change ADD score							
JIA	10	0.8 (1.9)	78	0.2 (1.8)	9	2.9 (4.6)*	
Controls	7	-0.7 (1.3)	85	0.2 (1.2)	9	1.1 (1.8)*	
Ulcerations - several times yearly or more							
Children - ECOHIS change ADD score							
JIA	6	-4.8 (4.6)	31	-0.3 (4.0)	7	-1.3 (2.1)*	
Controls	5	0.6 (3.6)	42	-0.5 (3.2)	3	-3.3 (3.5)	
Adolescents - Child OIDP change ADD score							
JIA	17	0.3 (1.2)	68	0.5 (2.6)	9	0.8 (2.2)	
Controls	19	0.3 (1.1)	68	0.1 (1.3)	14	0.8 (1.5)	
Orofacial pain the last 30 days							
Children - ECOHIS change ADD score							
JIA	6	-5.2 (3.1)	38	-0.2 (3.8)	3	-2.3 (4.5)*	
Controls	1	-7 (0)	53	-0.4 (3.2)*	0		
Adolescents - Child OIDP change ADD score							
JIA	16	-0.3 (2.2)	74	0.7 (2.5)	20	0.9 (3.2)	
Controls	6	0 (0.9)	93	0.3 (1.3)	3	-0.7 (2.1)	

One-way ANOVA: *p<0.05

Bonferroni post hoc analyses indicated the following (p < 0.05): Statistically significant differences in mean ECOHIS change by change score of satisfaction with tooth appearance for children in the JIA-group worsened vs improved; for change score of ulcerations in children with JIA worsened vs same; in both groups for change score of orofacial pain worsened vs same

Statistically significant differences in mean Child OIDP change by change score of pain during toothbrushing for adolescents in the control group worsened vs improved and for adolescents with JIA same vs improved.
