# Supplementary material for: Oral health-related quality of life, impaired physical health and orofacial pain in children and adolescents with juvenile idiopathic arthritis – a prospective multicenter cohort study
Source: BMC Oral Health. 2023 Nov 20;23:895. doi: 10.1186/s12903-023-03510-0 (PMC10662257; doi:10.1186/s12903-023-03510-0)
Supplement: Supplementary file 4 — Additional file 4: Mean OHRQoL ADD scores according to orofacial pain, CHQ physical scores, and disease activity (JADAS71). S4 Table 1. Mean OHRQoL ADD scores according to orofacial pain. S4 Table 2. Mean OHRQoL ADD scores according to CHQ physical scores. S4 Table 3. Mean OHRQoL ADD scores according to JADAS71. [file 12903_2023_3510_MOESM4_ESM.pdf]

**Additional file S4 – Mean OHRQoL ADD score according to orofacial pain, CHQ physical scores, and disease activity (JADAS71)**

**S4 Table 1.** Mean OHRQoL ADD scores according to orofacial pain

| | No pain | | Pain | | |
|---|---|---|---|---|---|
| | | **OHRQoL ADD score** | | **OHRQoL ADD score** | |
| | n/N | Mean (SD) [95%CI] | n/N | Mean (SD) [95%CI] | *p-value* |
| Children <12 years - mean ECOHIS ADD score | | | | | |
| First Visit - Orofacial pain ever | | | | | |
| JIA | 34/47 | 3.2 (4.2) [1.7-4.7] | 13/47 | 5.0 (3.9) [2.6-7.4] | *0.060[a]* |
| Controls | 48/54 | 2.0 (2.7) [1.2-2.7] | 6/54 | 2.7 (3.0) [-0.8-5.8] | *0.485[a]* |
| *p-value* | | *0.193[a]* | | *0.179[a]* | |
| First Visit - Orofacial pain last 30 days | | | | | |
| JIA | 40/47 | 3.7 (4.4) [2.3-5.0] | 7/47 | 4.0 (2.9) [1.3-6.7] | *0.219[a]* |
| Controls | 53/54 | 2.0 (2.6) [1.3-2.7] | 1/54 | 6 | *0.222[a]* |
| *p-value* | | *0.063[a]* | | *0.500[a]* | |
| 2-Year Follow-up - Orofacial pain ever | | | | | |
| JIA | 32/47 | 3.6 (4.0) [2.2-5.0] | 15/47 | 6.9 (5.2) [4.0-9.8] | ***0.009[a]*** |
| Controls | 51/54 | 2.2 (2.6) [1.5-3.0] | 3/54 | 9.0 (4.0) [-0.9-18.9] | ***0.005[a]*** |
| *p-value* | | *0.116[a]* | | *0.338[a]* | |
| 2-Year Follow-up - Orofacial pain last 30 days | | | | | |
| JIA | 37/47 | 3.7 (3.8) [2.4-4.9] | 10/47 | 8.3 (5.8) [4.1-12.5] | ***0.008[a]*** |
| Controls | 52/54 | 2.3 (2.6) [1.5-3.0] | 2/54 | 11.0 (2.8) [-14.4-36.4] | ***0.004[a]*** |
| *p-value* | | *0.056[a]* | | *0.546[a]* | |
| Adolescents ≥ 12 years - mean Child OIDP ADD score | | | | | |
| First Visit - Orofacial pain ever | | | | | |
| JIA | 63/110 | 0.5 (1.7) [0.1-1.0] | 47/110 | 2.0 (3.2) [1.0-2.9] | ***<0.001[a]*** |
| Controls | 87/102 | 0.5 (1.4) [0.2-0.8] | 15/102 | 0.7 (1.3) [-0.0-1.4] | *0.440[a]* |
| *p-value* | | *0.368[a]* | | *0.162[a]* | |
| First Visit - Orofacial pain last 30 days | | | | | |
| JIA | 79/110 | 0.6 (1.6) [0.2-0.9] | 31/110 | 2.7 (3.7) [1.3-4.0] | ***<0.001[a]*** |
| Controls | 99/102 | 0.5 (1.4) [0.2-0.8] | 3/102 | 0.3 (0.6) [-1.1-1.8] | *1.000[a]* |
| *p-value* | | *0.616[a]* | | *0.364[a]* | |
| 2-Year Follow-up - Orofacial pain ever | | | | | |
| JIA | 68/111 | 0.1 (0.4) [-0.0-0.2] | 43/111 | 1.3 (2.3) [0.5-2.0] | ***<0.001[a]*** |
| Controls | 85/102 | 0.2 (0.5) [0.0-0.3] | 17/102 | 0.8 (1.6) [-0.0-1.6] | ***0.015[a]*** |
| *p-value* | | *0.275[a]* | | *0.892[a]* | |
| 2-Year Follow-up - Orofacial pain last 30 days | | | | | |
| JIA | 84/111 | 0.2 (0.9) [-0.0-0.4] | 27/111 | 1.6 (2.5) [0.6-2.5] | ***<0.001[a]*** |
| Controls | 96/102 | 0.3 (0.8) [0.1-0.4] | 6/102 | 0.3 (0.5) [-0.2-0.9] | *0.381[a]* |
| *p-value* | | *0.293[a]* | | *0.486a* | |

[a]Mann-Whitney U test. OHRQoL =oral health related quality of life. ADD scores =additive scores. JIA =juvenile idiopathic arthritis. n/N =number observed/total number assessed. SD =standard deviation. CI =confidence interval. ECOHIS =early childhood oral health impaction scale. Child OIDP =child oral impact on daily performances. P-values <0.05 are marked in bold.

**S4 Table 2.** Mean OHRQoL ADD scores according to CHQ physical scores

| | Normal CHQ PhS | | Impaired CHQ PhS | | |
|---|---|---|---|---|---|
| | | **OHRQoL ADD score** | | **OHRQoL ADD score** | |
| | n/N | mean (SD) [95%CI] | n/N | mean (SD) [95%CI] | p-value |
| Children <12 years - mean ECOHIS ADD score | | | | | |
| First Visit | | | | | |
| JIA | 37/46 | 3.2 (3.6) [1.9-4.4] | 9/46 | 6.2 (5.5) [2.0-10.5] | *0.066*[a] |
| Controls | 52/52 | 2.1 (2.7) [1.3-2.8] | 0/52 | - | |
| *p-value* | | *0.106*[a] | | - | |
| 2-Year Follow-up | | | | | |
| JIA | 35/45 | 4.3 (4.5) [2.8-5.9] | 10/45 | 6.5 (5.1) [2.8-10.2] | *0.158*[a] |
| Controls | 50/54 | 2.5 (2.8) [1.7-3.3] | 4/54 | 4.0 (6.1) [-5.6-13.6] | 0.765[a] |
| *p-value* | | ***0.048***[a] | | *0.282*[a] | |
| Adolescents ≥ 12 years - mean Child OIDP ADD score | | | | | |
| First Visit | | | | | |
| JIA | 82/106 | 0.8 (2.1) [0.3-1.2] | 24/106 | 1.9 (2.7) [0.7-3.0] | ***0.023***[a] |
| Controls | 92/92 | 0.5 (1.3) [0.2-0.8] | 0/92 | - | |
| *p-value* | | *0.882*[a] | | - | |
| 2-Year Follow-up | | | | | |
| JIA | 71/99 | 0.1 (0.7) [0.1-0.3] | 28/99 | 1.3 (2.6) [0.3-2.3] | ***0.004***[a] |
| Controls | 78/79 | 0.2 (0.6) [0.1-0.3] | 1/79 | 0 | *1.000*[a] |
| *p-value* | | *0.339*[a] | | *1.000*[a] | |

[a]Mann-Whitney U test. OHRQoL =oral health related quality of life. ADD scores =additive scores. JIA =juvenile idiopathic arthritis. CHQ =child health questionnaire. PhS =physical scores. n/N =number observed/total number assessed (CHQ missing in 6 participants with JIA and 12 controls). ECOHIS =early childhood oral health impaction scale. Child OIDP =child oral impact on daily performances. SD =standard deviation. CI =confidence interval. P-values <0.05 are marked in bold.

**S4 Table 3.** Mean OHRQoL ADD scores according to JADAS71

| | JADAS71 <1 | | JADAS71 ≥1 | | |
|---|---|---|---|---|---|
| | | **OHRQoL ADD score** | | **OHRQoL ADD score** | |
| | n/N | mean (SD) [95%CI] | n/N | mean (SD) [95%CI] | p-value |
| Children <12 years - mean ECOHIS ADD score | | | | | |
| First Visit | 14/47 | 2.1 (3.6) [0.1-4.2] | 33/47 | 4.4 (4.2) [2.9-5.9] | ***0.038***[a] |
| 2-Year Follow-up | 17/46 | 3.5 (3.8) [1.6-5.5] | 29/46 | 5.4 (5.1) [3.5-7.3] | *0.267*[a] |
| Adolescents ≥12 years - mean Child OIDP ADD score | | | | | |
| First Visit | 40/107 | 0.3 (1.0) [-0.1-0.6] | 67/107 | 1.5 (2.7) [0.8-2.1] | ***<0.001***[a] |
| 2-Year Follow-up | 40/103 | 0.2 (0.6) [-0.0-0.3] | 63/103 | 0.6 (1.6) [0.2-1.0] | *0.166*[a] |

[a]Mann-Whitney U test. OHRQoL =oral health related quality of life. ADD scores =additive scores. JIA =juvenile idiopathic arthritis. JADAS =juvenile arthritis disease activity score. n/N =number observed/total number assessed (CHQ missing in 6 participants with JIA and 12 controls). ECOHIS =early childhood oral health impaction scale. Child OIDP =child oral impact on daily performances. SD =standard deviation. CI =confidence interval. P-values <0.05 are marked in bold.
